# How is return on investment from quality improvement programmes conceptualised by mental healthcare leaders and why: a qualitative study

**DOI:** 10.1186/s12913-023-09911-9

**Published:** 2023-09-19

**Authors:** S’thembile Thusini, Tayana Soukup, Kia-Chong Chua, Claire Henderson

**Affiliations:** 1 King’s College London, London, UK; 2 Imperial College London, London, UK

**Keywords:** Return on investment, ROI, Quality improvement, QI

## Abstract

**Background:**

Return on Investment (ROI), whereby the ratio of costs to benefits is assessed, is encouraged in-order to justify the value of Quality Improvement (QI) programmes. We previously performed a literature review to develop a ROI conceptual framework for QI programmes. We concluded that, QI-ROI is conceptualised as any monetary and non-monetary benefit. In the current study, we explored if this finding is shared by mental healthcare leaders. We also investigated the stability of this conceptualisation against influencing factors and potential for disinvestment.

**Methods:**

We performed qualitative interviews with leaders in an NHS mental health organisation. There were 16 participants: nine board members and seven senior leaders. The interviews were held online via Microsoft Teams and lasted an hour on average. We performed deductive-inductive analysis to seek data from our initial ROI framework and any new data.

**Results:**

We found that in mental healthcare, QI-ROI is also conceptualised as any valued monetary and non-monetary benefits. There was a strong emphasis on benefits to external partners and a de-emphasis of benefit monetisation. This conceptualisation was influenced by the 1) perceived mandates to improve quality and manage scarce resources, 2) expectations from QI, 3) health and social care values, 4) ambiguity over expectations, and 5) uncertainty over outcomes. Uncertainty, ambiguity, and potential for disinvestment posed a threat to the stability of this conceptualisation but did not ultimately change it. Health and social care values supported maintaining the QI-ROI as any benefit, with a focus on patients and staff outcomes. Socio-political desires to improve quality were strong drivers for QI investment.

**Conclusion:**

Mental healthcare leaders primarily conceptualise QI-ROI as any valued benefit. The inclusion of externalised outcomes which are hard to attribute may be challenging. However, mental healthcare services do collaborate with external partners. The de-emphases of benefit monetisation may also be controversial due to the need for financial accountability. Mental healthcare leaders recognise the importance of efficiency savings. However, they raised concerns over the legitimacy and utility of traditional ROI as a tool for assessing QI value. Further research is needed to bring more clarity on these aspects of the QI-ROI concept.

**Supplementary Information:**

The online version contains supplementary material available at 10.1186/s12913-023-09911-9.

## Introduction

The need for higher quantity and quality of mental health services has always been paramount [1, 2]. The COVID-19 pandemic has added to this need due to the escalated global medical, social, and financial distress [3, 4] at a time when mental health services are grappling with chronic funding gaps [5–7]. In 2021, the United Kingdom (UK) National Health Service (NHS) ring-fenced over £2.3 billion for mental health services [8]. Some of this will likely be spent on Quality Improvement (QI) programmes. Unlike small, localised QI projects, QI programmes combine a variety of strategies in local, national or international efforts to systematically improve quality and efficiency of organisations and or healthcare systems as a whole [9, 10]. As healthcare resources are limited, leaders must make and justify investment allocation decisions. The World Health Organisation (WHO) has recommended Return on Investment (ROI) as a tool to justify investments in mental healthcare programmes [11].

ROI can forecast financial value (e.g., in business cases), or evaluate a programme ex-post [12]. Outcomes are converted to money (monetised) so as to assess the financial gains that a programme can or has yielded [12]. To do this, a financial value of a benefit must be directly or indirectly determined using financial approximates (proxies) [12, 13]. Traditionally from accounting, little is known about ROI in healthcare. As such, there is a need for an operational definition of ROI in mental healthcare QI [14]. We recently performed a systematic literature review where we studied ROI as a concept of returns or benefits from QI. This led to two studies; the first [15] describes our ROI concept analysis, development of the QI-ROI concept and its framework. We then further developed the framework by adding benefits that make up QI-ROI [16]. In the current study, we explored if this conceptualisation is shared by mental healthcare leaders. We also explored the factors that influence their conceptualisation of ROI.

Currently, there is no standardised QI governance tool to guide QI investment allocation decisions [17]. This hinders effective assessment and communication of QI value in mental healthcare and healthcare as a whole [18, 19]. However, before these challenges can be addressed, it is crucial to learn what the meaning of ROI in mental healthcare organisations is and why. This understanding is needed to help provide practical support to leaders in their QI investment/disinvestment decision-making processes. This is important as quality of care and cost of care are central to the goals of both healthcare provision and QI programmes [20, 21].

ROI is closely related to QI effectiveness (goal achievement) as achievement of stated QI programme goals is part of QI value. QI effectiveness has so far been found to be inconclusive [22–28]. However, the review preceding this study found that most healthcare leaders and other stakeholders saw QI benefits regardless of its effectiveness, for example in lessons learnt from failed attempts [16]. QI cost-effectiveness and cost-efficiency (achieving the best outcome for the least cost, and without waste) is also a part but not a complete picture of QI value [29]. A programme may be cost effective and efficient but fail to provide value in other important organisational aspects [30]. Therefore, a more comprehensive view of ROI is needed. This requires a deeper understanding of the meaning of ROI in mental healthcare. To this effect, we employed institutional theory as our study’s underpinning theory.

Institutional Theory is the overriding theoretical base for this study, due to its explanatory power regarding organisational behaviour and reasoning [31]. Institutional theory encompasses various connected, sometimes contradicting theories. Traditionally, institutional theory focused on organisational outcomes and their causes [32, 33]. The newer neo-institutional theory asserted that organisations are governed by taken-for-granted norms and values, often to economic detriment [34]. Faced with institutional pressures, organisations are said to either blindly follow norms, be coerced to complying, or mimic other organisations. This then leads to isomorphism (homogeneity) and promote social legitimacy rather than efficiency [34]. However, others argued that organisational actors do have influence [35, 36].

Through language in discourse (discourse theory) or rhetoric (rhetoric theory), organisational actors (re) create, communicate, and legitimise new meanings [37, 38]. Discourse is a system of knowledge, meaning-making, and communication which is greatly influenced by power relations in a context [37]. Rhetoric is related to the use of language to manipulate a message towards a desired end [38]. Discourse and rhetoric are related to a branch of institutional theory called Institutional Logic [39]. An institutional logic is a set of organising rules and norms that influence meaning-making and reasoning in a context [38, 40]. Institutional ‘entrepreneurs’ are said to engage in strategies to create and change prevailing logics [41]. These theories align with Institutional Work, a theory which highlights role dependent actions and meanings [41].

Related to institutional theories are the Stakeholder [42], and Stewardship theories [43]. The stakeholder theory denotes that leaders strategically engage stakeholders (individuals that impact or are impacted by an organisation) to incorporate broad social values [42]. The stewardship theory states that autonomous leaders are more likely to be intrinsically motivated, geared towards mutual gain and collaboration [43]. All these theories acknowledge the institutional context within which meaning is socially constructed. We assess which of these theories are reflected in our findings. This deepened our understanding of why QI-ROI was conceptualised a certain way, and hence the antecedents of the QI-ROI concept.

The main aim of this study was to ascertain the extent to which the conceptualisation of QI-ROI as any benefit is shared by mental healthcare leaders. Our second aim was to further develop the QI-ROI concept and its conceptual framework for mental healthcare. The study addressed the following research questions:

1. How is ROI from QI programmes conceptualised by mental healthcare leaders?
2. What influences that conceptualisation?
3. How is that conceptualisation impacted by a potential for QI disinvestment?

## Methods

### Study setting

All participants were employed by an NHS Trust, a mental health care provider in London. This setting has been engaged in quality improvement using QI methodologies since 2016.

### Study design

We chose an interpretive qualitative design as this approach helps provide insights into a complex world where participants’ reality is assumed to be socially constructed [44]. This is a stakeholder-centred methodological approach, where ‘reality construction’ occurs amongst the participants, and between the researcher and participants [44, 45]. This invokes the concept of double hermeneutics where multiple views reflect context-focused collective meaning-making [45].

### Participants

Eligible participants were the board members as QI investors (e.g., Chief Financial Officer) or senior managers as influential leaders (e.g., Clinical Directors). To be included, participants had to have experience in QI activities (investment, implementation, evaluation), and have internet access. Leaders not influential in QI implementation, evaluation and investment were to be excluded. Our target sample was 15, which we deemed both feasible and sufficient to gain insights on the QI-ROI concept at high leadership level. We employed purposive sampling to target suitable informants. Potential participants were identified by and initially approached by the Trust’s QI Director, and co-author CH before being engaged by first author, ST. Twenty four potential participants were approached.

### Procedures

The following procedures were carried out by ST. Potential participants were sent formal invitations and participant information sheets (PIS) which detailed the study aims and objectives. Participants were informed about the study as part of ST’s PhD project. They were given an average of two weeks to consider the study and ask questions. Written consent from each participant was obtained prior to their interview. Individuals interviews were performed online via Microsoft (MS) Teams [46]. The topic guide (Supplementary file 2) contained semi-structured questions to help explore the results from the systematic review and collect new data [47]. Interviews took place between December 2021 and January 2022 and lasted about an hour each. Interviews were recorded and transcribed using the MS Teams. During transcription, participants personal details were deleted for anonymity. Data were moved to NVivo Release 1.6 [48], securely stored and managed in encrypted, and password protected King’s College London computer. All data from MS Teams were deleted. A COREQ [49] checklist for our study can be found in Supplementary file 1.

### Data analysis

We analysed the data using Framework Analysis, a deductive approach that uses an existing framework to deduct specific data from a dataset [50]. Combined with thematic analysis, this approach allowed us flexibility to induct new data [51, 52]. Our analysis had two phases: Phase I (the deductive phase), which included steps 1) developing a codebook based on the conceptual framework, and 2) testing the codebook against the existing framework and literature and adjusting codes as needed. Phase II (the inductive phase) entailed step 3) re-familiarising with interview data, 4) generating initial codes, 5) applying the codebook and identifying any additional codes, 6) connecting codes, 7) identifying themes, 8) re-checking themes, and 9) reporting study findings. Our data analysis framework can be found in Supplementary file 3. Our codebook and additional exemplary quotes can be found in Supplementary file 4.

## Results

Of the 24 potential participants approached, 16 took part in the study: nine board members and seven non-board directors were interviewed. Eight leaders were unable to partake due to their work schedules. We arranged themes from the data according to our three research questions: ROI conceptualisation (question 1), Influencing factors (question 2), and Disinvestment potential (question 3). ROI conceptualisation is the leader’s mental abstractions of QI-ROI as represented by how the leaders’ defined and described ROI. Influencing factors were those that directly influenced the consistency of ROI conceptualisation. Disinvestment potential relates to how all factors affect QI investment decisions, and how in-turn the potential for disinvestment impacts the stability of the conceptualisation. This indirectly reflects on the consistency of the QI-ROI concept as collective investment decisions may be based on the Trust’s prevailing ROI conceptualisation.

### ROI conceptualisation

Mental healthcare leaders predominantly described ROI as any valued benefit that directly or indirectly contributes to the fulfilment of their organisational strategy. Here, ROI was associated with quality where the improvement in quality as demonstrated by desired outcomes was seen as a return (on investment). Valued QI benefits included patient outcomes, staff outcomes, financial outcomes, organisational development, and external outcomes for healthcare systems and societies. Financial benefits were seen as secondary to other outcomes.

“[we are] not looking at the money but actually we're driving the money from the quality of the service…high quality, ultimately costing us less and, giving us more scope for investment and innovation”. Participant 7.

A few participants associated ROI with the cost of care. In this description, the leaders focused more on costs (and investment) and less on the benefits or returns. This suggested ROI conceptualised more as a cost-saving or cost-management tool. For example, some leaders discussed using QI to prevent future high-cost care, maximise benefit, and thus save money.

“…saving money, achieving a sustainable organisation because we're working in an era of … severe constraints…Because it's public money, we should make sure that it's used to maximise the benefit of the people that we serve”. Participant 3.

### Influencing factors

Influencing factors consist of five interlinking subthemes. These are: healthcare mandates, values, expectations, ambiguity, and uncertainty. These factors reflected the complex nature of QI programmes as well as the complexity of mental healthcare.

#### Healthcare mandates

Participants strongly suggested that their conceptualisation of ROI was anchored on what they saw as their mandates as mental healthcare leaders. Mandates were often external in their nature, for example through national quality frameworks (e.g., restraint reduction) or fiscal targets. The main QI mandate was seen as improving the quality of services. Indirectly, managing scarce resources was an important QI mandate that can eventually lead to saving costs. In addition, were perceived obligations towards patients, staff, societies, the organisation, and partners. Leaders also expressed internal aspirations such being the best service provider.

“I would like to see reduction in terms of money, but I think the quality aspects of supporting people in their lives and their recovery journey is a really valid way to show that that investment’s been worthwhile. Participant 10.

A few leaders referenced the organisation’s overall strategy on environmental sustainability. However, the main concern for most participants was the sustainability of QI programmes and outcomes. Sustainability was seen to be related to supporting and embedding new practices. Some participants were concerned about the organisation’s ability to cater for future patients. These participants felt that this required the organisation to be self-sufficient and sustainable. In this regard, QI was used to improve organisational efficiency and productivity rather than generate profits. This was expected to help free-up and redeploy resources where most needed.

“…when I talk about financial benefits, I talk about how we then reinvest that to make us a more sustainable service for the future, knowing that we've got increased demands often in decreasing capacity or capacity that's unsustainable …”. Participant 2.

Managing scarce resources, improving quality, and sustainability meant seeking cultural transformation. Through QI, internal and external cohesions or collaborations were encouraged. Internally, this entailed improving team-working and engaging patients, whilst externally cohesions and collaborations entailed relationships with external partners and communities. Cohesion and collaboration were sought through co-production, enhanced communication using a shared language, and sharing leadership. There was a desire to disseminate insights both internally and externally to the organisation. The assumptions were that through collaboration, benefits could be maximised, spread more quickly, and sustained. QI collaboration was expected to improve efficiency and avoid “reinvention of the wheel”.

“…the benefits of that for the Trust of doing it this way, is that where improvements are made in a pilot, they can then be rolled out … so that we're not reinventing the wheel, which is resource heavy”. Participant 9.

Leaders often made links between different outcomes. Particularly, improved staff skills were seen as central to achieving both patient and financial outcomes, as well as system-wide outcomes. Therefore, some QI investment was also keenly directed towards staff outcomes.

#### Values

Mandates determined QI’s main goals and objectives and provided rationales for pursuing QI. These mandates were often explicitly and implicitly expressed through values. Some values were extrinsic, for example, QI was a means to manage economic pressures from rising service costs and demands. However, leaders predominantly expressed intrinsic motivation to improve service outcomes. Participants primarily viewed ROI through personal, professional, and or organisational values. Extrinsic values were applied within the framework of intrinsic values.

“I think when you see things like quality adjusted life years and monetised outcomes that can be used within health, I think that makes the hairs on lot of clinicians’ backs go up. …I think people can feel very uncomfortable with those monetised outcomes”. Participant 11.

Health and social care perspectives drove the predominant intrinsic values. Such values prioritised clinical and social agendas such as upholding human rights and justice. This included value-based healthcare where any outcome that matter to patients is favoured. Here, outcomes included helping individuals improve their personal, social, and work lives.

“By reducing restrictive practices, we're respecting people’s human rights. We are improving their wellbeing. We are increasing the chances of their recovery. There's a very human quality there that you can't monetise”. Participant 9.

There was recognition that the traditional ROI is meant to encourage fiscal responsibility. However, leaders collectively rejected the notion that only monetisable benefits should count as ROI in mental healthcare. Even though some named benefits were monetisable, most leaders’ ROI concept was focused on the benefit itself, and not the monetised version.

“…we could get an ROI actually to pick it apart and get what the cost is, and cost saving is. It's not really worth it for the investment…It doesn't matter what we're getting in return [on investment]. The important thing is the, the actual outcome”. Participant 8.

For some, their role meant conceiving ROI as primarily measurable outcomes. This meant treading a fine line between a broad based ROI and the econometric traditional ROI. This modification indicated a lean towards financial ROI (which can be measured) and or a tension between economic and healthcare values. Framing messages ‘correctly’ was important in managing this tension and fulfilling multiple obligations.

“We're running healthcare delivery organisation using the best of business practice and principles. It's a very different way of describing it. And it's a fundamental tension when you have a board where people talk about ROI and cash releasing, savings and flow…I mean, it's blindingly obvious if our patients don't end up in deep poverty, they are not going to relapse as much”. Participant 2.

Quality of care was not described in financial terms. There was a desire to avoid financial focus at the expense of desired goals like staff and patient outcomes. For many, a balanced ROI approach that combines both financial and non-financial outcomes was crucial.

“CQC (Care Quality Commission) will rate on the quality of services. But if their finances were in a mess, they wouldn't be able to be an outstanding [organisation]. It is about making sure that you're getting a good return on investment, both in terms of being financially sustainable but delivering first class services”. Participant 5.

#### Expectations

Participants’ expectations were driven by mandates, perceived obligations, and values. The QI outcomes described as ROI aligned with what QI was expected to do or enable. All participants described QI as a systematic way to improve the quality of healthcare. QI was seen as a mechanism for staff empowerment, diagnosing problems, understanding systems, identifying, and testing solutions, as well as avoiding excessive waste by abandoning failing attempts early.

“…it's starting from a position of somewhat kind of helplessness about what they [staff] could do about the situation…” Participant 13.

The aim was to then embed, roll-out, and sustain improvements. In both instances (failure or success), lessons were to be taken forward and shared with other teams within the organisation or with partners. The ultimate objectives were sustained improvements in desired outcomes.

“We pursue quality improvement programs to embed in the organisation, approaches which deliver better quality care”. Participant 3.

#### Ambiguity

Expectations often varied amongst participants, depending on their QI function knowledge and or buy-in, as well as influence of others.

##### QI function knowledge and buy-in

QI function knowledge was strongly related to a leader’s role, QI training, experience, and or proximity to QI programmes. The closer in proximity to QI programmes, the more QI training and experience, the broader the view of the QI-ROI concept. This was accentuated in those that were ‘bought-in’ into the QI methodology. Those not bought-in were sceptical about function, outcomes, and causality. Some were concerned about assumptions by others that QI can solve any problem. Negative experience limited expectations or forced adjustments to be more ‘realistic’. Personal experience with QI enabled more nuanced understanding of QI benefits beyond achieving programme goals in terms of organisational and system-wide outcomes. This enabled clearer expression of both immediate and long-term QI outcomes, and or broadened views on what QI is most suited for.

“I think some people, especially in a healthcare setting, would see quality as patient care, for an individual patient. Some might see as your ability to treat your population, so that's more of a performance element of quality, and I would include how you utilise your resources sustainably to maximise quality” Participant 1.

##### QI Success vs QI failure

Some saw QI effectiveness as encompassing outputs e.g., diagnosing process issues, or hard outcomes e.g., achieving set goals. Including softer benefits broadened the view of QI-ROI. Perceiving QI as a continuous incremental methodology also broadened the QI-ROI concept. Here, different benefits were perceived throughout what was seen as a “QI journey”. The assumption was that QI projects can be aggregated to unlock organisation level outcomes that improve overall performance. The collective perception was that not all QI is successful.

“Rather than spend a year setting something up and then failing, can we set up in a week and fail quickly so that we know, what's not working quickly. But in doing so, not discounting it, giving the chance to properly fail”. Participant 15.

##### Intervention vs implementation failure

QI-ROI was seen to be related to intervention and implementation failures. Intervention outcomes for some programmes were perceived to have been mostly positive. However, poor initial implementation, embedding, rolling-out, scaling-up, spread, dissemination, and sustainability were most frequently associated with failure to obtain QI-ROI. Deciphering the exact failure or cause, or even it was indeed a failure however seemed challenging.

“One of my frustrations would be that I think it's hard to see the return on investment from QI at the moment. …there are promising elements of what we have been trying to do, …It looks it looks like it works…, but never rolled out. So, is that a success of QI, or is that a failure of QI? Hard to say!”. Participant 1.

##### QI evaluation; how, what, and when to measure

Some desired outcomes were deemed neither measurable nor monetisable (e.g., well-being). This then created a dilemma of how QI-ROI should be assessed. Participants stated that a compromise can be reached through using proxies and or adding narratives to detail qualitative benefits. QI and thus ROI measurability (and monetisability) was deemed necessary by some. However, the lack of skill and or infrastructure was a challenge. The measurement challenges also included the ROI methodology itself. These concerns co-existed within some participants.

“I think we're fairly unsophisticated when it comes to thinking through the stuff that's harder [to measure], partly because we probably strapped for resource, and we don't have the people who have the time to think through bit more of the sophisticated proxy measures that we might like to use”. Participant 4.

I think that social impact is really important, and I don't believe that financial only ROI is sophisticated enough”. Participant 4.

There was differences of opinions as to when to measure QI outcomes. Some felt there was a misalignment between expectations of immediate results and the ability of organisational and QI processes to deliver immediate results. Most participants asserted that QI benefits do not show themselves in the immediate period, they come in phases. Some outcomes such as problem identification and diagnosis may be immediate, others such as patient outcomes may be intermediate, whilst sustainability and cost-saving were seen as long-term outcomes.

“If QI really is working, then then those outcomes should be being delivered. So, year on year…but I think if QI was really working you should be able to identify the next problem much more easily too”. Participant 8.

For some, QI was expected to be continuous and incremental and then aggregate to transformation (a complete change). However, some participants did not see QI as a tool for transformation. They felt QI can contribute to transformation, and top-down measures were needed for organisational transformation. This ambiguity co-existed within some participants.

“…[QI] it's a way of being able to know what it is we want to strategically deliver in the medium to long term and then use our methodology to start to make incremental changes that we know will aggregate up to that big change.” Participant 12.

“…all you do is just make those incremental changes, but never changed the system for the future. So, you've already failed before you started, because yes, you can improve processes to a particular percent or degree actually without really changing the whole value chain…you won't make systemic longstanding change”. Participant 12.

##### Influence of others

Some scepticism was related to the influence of others within and outside the organisation. Internally, trusted colleagues’ negative perception of QI or QI effectiveness narrowed others’ concept of QI-ROI due to the limited perceived benefits. There were also concerns regarding what was seen as faulty assumptions by others regarding what to expect from QI. External influential sources included literature, health economists, and politics. Lack of evidence of QI effectiveness in literature limited expectations and created scepticism. Awareness of ROI as monetised benefits from health economics literature was taken into consideration. Political and economic expectations caused some to frame their views of ROI as monetisable benefits, without fundamentally changing their conceptualisation of ROI as any valued benefit. Some modified their QI-ROI concept to fit context (e.g., economic, political contexts). There was a belief that economists also value intangible outcomes (that cannot be measured or monetised).

“I would say if I was talking to an economist who believed in intangible assets, which I think many of them do, then I would kind of take that position. But I think someone who purely wants to look at ROI where there would be something that stacks up that I can count, then I probably [would] deviate a little bit from that position”. Participant 16.

##### QI philosophy; theory and practice

There appeared to be various interpretations of the same QI concepts, for example the QI trial-and-error principle and QI effectiveness above. Ambiguity was also seen as resulting from competing goals. However, some indicated that the issue was less of ambiguity, but more of a dissociation between QI theory and practice.

“There's a difference between the concept, which absolutely remains fundamental, and the mechanism for delivery. You have to hold them separately…”. Participant 1.

The apparent contradictions appeared to have the effect of QI working against itself by producing results that are contrary to the QI philosophy and principles being promoted. For example, although QI is by principle a bottom-up approach, some as experienced QI as a top-down approach. Others found that QI could create silos rather promote collaboration. Others found that a shared language and communication was not as desired. Others felt discomfort at the ‘insistence of the use of 'Guru’ concepts. Structuring of QI was seen by some as a potential threat and counterintuitive to innovative thinking, as well as flexibility and adaptability.

“I've observed in the organisation, for example, as we've been running kind of three-monthly improvement cycles and testing interventions, and it's become sort of almost psychotic with no real thinking as to what's the big overall issue”. Participant 16.

#### Uncertainty

##### Lack of dedicated ROI evaluation tool

There were uncertainties and scepticism regarding whether QI does live up to expectations. The board was said to have the responsibility to manage uncertainty and assessment of ROI. At the board level, QI-ROI was expected to be demonstrated by improvement in key performance measures within the organisation’s integrated quality framework. Some leaders provided specific data on ROI links to integrated quality framework. For example, staff and patient surveys as part of understanding the QI-ROI from collective QI programmes. For others, the ‘sense’ of QI-ROI was through intuition, first-hand knowledge, or dissemination.

“How can we measure the improvement compared with the investment that it's taken, and we have seen significant moves forward in some areas …”. Participant 7.

There was recognition that the ideal of perfect information was impossible. What leaders sought, was enough information to make a decision. Sometimes that meant accepting the reality of inability to provide definitive proof. Some information was seen as better than none.

“…some things you know we have to accept; we just can't measure”. Participant 5.

##### Uncertainty due to poor communication

Some saw ambiguities and dilemmas as opportunities to engage in communication over QI-ROI. Some participants commented on the poor communication within the organisation regarding what QI has or has not achieved. This appeared to worsen overall uncertainty over QI outcomes and therefore QI-ROI. Some participants noted that the focus on COVID-19 had, however, significantly impacted routine communications. At times poor communication was related to avoidance of the subject altogether due to the discomfort surrounding ROI.

“…the challenge is we are really bad on return on investment articulation and measurement, and I think we are deliberately bad on it. And the reason we are deliberately bad on it is that it’s an uncomfortable place to be. [A practitioner] very rarely wants to sign up to it, because then they actually have to deliver on the aspirations”. Participant 12.

##### Uncertainty over causality

Most participants indicated that organisational complexity challenged what can be realistically expected or causally linked to QI programmes. Participants expressed that in mental healthcare, costs and benefits may be shared with external partners, making ROI harder to assess.

“…we're now doing much more collaborative work with our partners and… so you know in terms of being able to assess what our role was and financially say, that this is a bit we did is much more tricky to do”. Participant 10.

Some felt that as the QI investment is fixed, and as such, QI-ROI should be based on actual outcomes of QI programmes (e.g., improved safety), rather than monetised outcomes (ROI).

“We don't think it's worth us going away and working that part out…that's not quite the way we think about it, so we wouldn't do the financial or economic analysis on and every program. Absolutely not. We're much more focused on the program outcomes for each piece of work [because] we have a fixed investment into QI”. Participant 8.

Some participants indicated that QI was already embedded and part of everyday business. However, some appeared unable to disentangle QI principles or philosophy from everyday or other ways of innovative working. It appeared difficult to tell when QI is embedded, and or when QI was not a factor in how things are done or thought of.

“I don't think I once thought about QI in that time or anybody did, we did use data quite a lot and most organisations now use run charts so that I wouldn't say that's particularly QI it was a QI-centric decision”. Participant 14.

This was also apparent when discussing QI’s performance during the COVID-19 pandemic. There were differences in opinions as to how QI performed during the pandemic. Others explained this to be a result of QI being already embedded within the organisation. Some thought that an indication of this embedding could be the speed of implementation or problem diagnosis. Some participants felt that this was achieved during this pandemic.

“…when COVID hit [they] were absolutely phenomenal, they've never done this before…and boy, did they make it happen! You know, it's incredibly impressive what they did, and it did happen quickly, so you know, that's the point”. Participant 9.

### Disinvestment potential

The influencing factors above also affected attitudes towards QI investment and disinvestment. The view of most participants was that QI methodology is known to be effective, as demonstrated by other industries or other mental healthcare organisations. As such, QI was generally supported. Although some questioned the QI methodology, investment into improving quality was seen as a necessity, an obligation, and a fundamental philosophical organisational position. In the face of ambiguities, dilemmas, and uncertainties; patience, compromise, and tolerance were exercised. Investment decisions were tailored to expectations based on what was seen as the reality.

“…sometimes you make decisions based on the fact that you won't get any of this. You know you won't get any financial returns. Sometimes you make it based on the fact that actually you’ll save”. Participant 4.

For some participants, financial pressures meant that some form of proof was essential to continue QI investment and support. Failure to provide proof created reluctance towards future QI investment. Some were concerned that this potentially creates a vicious cycle where uncertainty over ROI may lead to disinvestment, and resource disinvestment may lead to more failure and or further uncertainty. Thus, QI was viewed as a risky investment by some. There were also concerns that unwarranted investment into QI could result in a ‘locked-in’ state.

“I think there's sometimes there is a risk in not doing that that you get locked into something that feels right, and for the general good…”. Participant 7.

A participant described this as an issue with investing into the concept, and not the actual daily QI realities. Most participants expressed that QI failure was strongly linked to teams and organisations failing to implement and support QI. There was an awareness that organisational-level challenges such as board governance, resources, as well as QI team governance determined QI success. QI was seen as everybody’s business.

“I've seen some things where people don't implement it right. They don't provide the support and training for staff to do it…, you know you can implement it really badly. Just make it a process that people feel like they're going through. They don't feel any benefits for themselves….and you know you'll fail on it…” Participant 6.

Despite uncertainties, leaders appeared unwilling to disinvest from QI. Instead, they preferred re-examination and redesigning of improvement efforts using different tools or approaches within the QI methodology.

### Summary of the themes

The above findings reflect the three themes based on research questions: the conceptualisation of QI-ROI, its influencing factors, and the impact of potential for disinvestment on the concept. Influencing factors had five subthemes: the perceived QI mandates, values, expectations, ambiguity, and uncertainty. There appeared to be a tension and mutual dependency between improving quality and managing scarce resources; the finances and other resources helped improve quality, and improved quality helped manage scarce resources. Ambiguity and uncertainty appeared intricately linked in a self-reinforcing cycle. Different ambiguities led to uncertainty, and uncertainty caused ambiguity. Institutional complexity added to the sense of uncertainty over both costs and benefits from QI. Ambiguity over expectations and uncertainty over QI outcomes posed a threat to the stability of the QI-ROI concept.

The inability to measure and or monetise some valued benefits constituted lack of QI-ROI objective proof. For some, this caused a rejection of non-financial benefits as a legitimate part of ROI, although they remained legitimate QI benefits. The rejection of non-financial benefits appeared to be based on what the traditional ROI warrants. Alternatively, uncertainty due to the inability to measure and or monetise valued benefits emerged as a strong factor in rejecting traditional ROI. Most leaders saw it inconceivable not to include certain valued benefits due to their non-monetisability. In this context, a valued benefit was any that contributes to achieving desired strategic goals. Crucially, leaders were more concerned about measurability (and ‘attributability’) of outcomes than monetisability of outcomes. The attitudes towards QI disinvestment in the face of uncertainty indicated a stability of QI-ROI as any valued benefit.

**Fig. 1:**
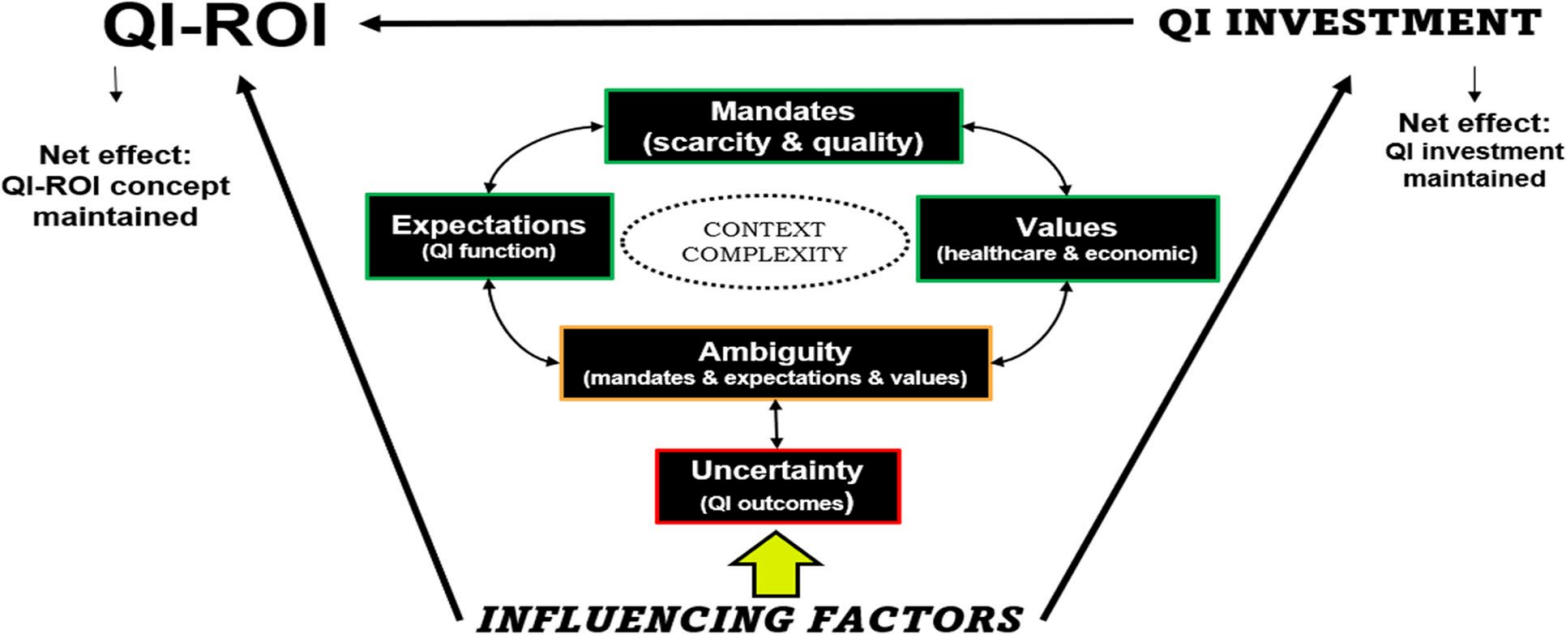
Shows the QI-ROI concept and its influencing factors

The net effect was the shifting of the ‘dial’ within the monetary-nonmonetary QI-ROI dimension, with a bias for non-monetary benefits. Some benefits would be included, excluded, or not even considered. Nonetheless, the QI-ROI concept was maintained as any valued monetary and non-monetary benefit, and the QI investment was also set to continue. The perceived mandates and values played a significant role in defining the QI-ROI concept as any benefit. Particularly, the focus on quality had a strong positive influence on maintaining a comprehensive QI-ROI concept. Concerns about scarce resources caused modifications to include financial benefits and view of ROI as a cost-saving tool. The themes are summarised in Fig. 1. Additional quotes, codes, and themes can be found in Supplementary file 4.

## Discussion

The main objective of this study was to explore the extent to which the conceptualisation of QI-ROI indicated by our prior systematic literature review [16], was shared by mental healthcare leaders. This study found that participants also conceptualised QI-ROI as any valued benefit that contributes to the fulfilment of their organisational strategy. The QI-ROI concept was represented by improved quality in certain internal and external aspects of the organisation. In conceptualising QI-ROI this way, the leaders followed a health and social care logic that prioritises patients’ clinical and social outcomes over monetary outcomes. This differentiates QI-ROI from traditional ROI [12]. Also supported in this study, was a de-emphasis of benefit monetisation, valuing hard to measure and comprehensive internal and external benefits. Thus, this conceptualisation of QI-ROI has challenges, including ambiguities and uncertainties.

The were differences in opinions that gave an organisational level ambiguity over the specifics of QI-ROI, e.g., definitions of QI success and failure. Expectations from QI provided a framework for how ROI from QI was conceptualised. Ambiguities over expectations appeared to lead to ambiguity over QI success and failure. For example, this study indicated that QI can be valued regardless of failing to achieve its intended goals. Further, success at unit or project level was associated with achievement of goals (intervention effectiveness), whilst failure at programme or organisational level was portrayed as implementation failure. In the following section, we discuss our updates on the QI-ROI conceptual framework, and these controversies surrounding this QI-ROI concept.

### The QI-ROI conceptual framework

**Fig. 2:**
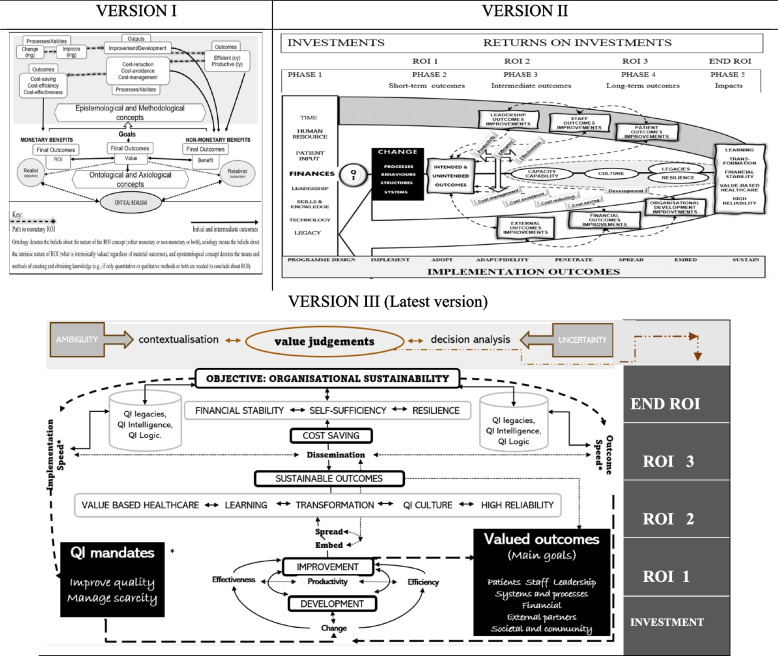
Shows the revised QI-ROI Conceptual Framework (versions I-III). Versions I & II are re-published with permission by Thusini et al. [15, 16] respectively. Version III shows the new additions* implementation outcomes  QI journey  main process outcomes  collateral outcomes

Conceptually, the current study has indicated identical QI-ROI components with the previous literature review. QI contribution to organisational strategies e.g., value-based healthcare (VHBC), transformation, and resilience was also found to be important here. As such, only minor changes were made on the QI-ROI conceptual framework (Fig. 2). The main goals were predominantly presented as patient and staff outcomes. However, other internal and external benefits were also valued. Two main new outcomes emerged as important, an interest in organisational sustainability, and the speed of diagnosis of problems and solutions. QI was said to be driven by two main mandates, to improve quality and manage scarce resources. Thus, new additions in the QI-ROI conceptual framework are the QI mandates as the starting points alongside investment, organisational sustainability as the ultimate objective, and speed as an emerging valued implementation outcome.

The revised framework therefore illustrates the following: at initial implementation, change ensues. Either as a specific strategy or part of doing QI, development occurs (e.g., staff development), and collaboration with partners (e.g., other teams) may occur. This could lead to improved productivity, effectiveness, and efficiency. This may lead to improvements and good practice which must then be embedded, spread, and or disseminated. In the process, QI may contribute to other strategies such as VBHC and transformation. Eventually, organisations may save costs, become financially stable, self-sufficient, resilient, and sustainable. In addition, the framework also indicates that the inherent ambiguities and uncertainties surrounding QI-ROI may be minimised through focused contextualisation, decision analysis, and value judgements [53–56].

This process (the QI journey) occurs through phases, and gains are re-invested back to the organisation and its stakeholders. Initial investment occurs in phase 1, immediate outcomes maybe gained in phase 2 (ROI 1), intermediate outcomes in phase 3 (ROI 2), longer-term outcomes in phase 4 (ROI 3), and some outcomes may be sustained (end ROI or ROI 4). These phases correspond with four main QI-ROI components: development and improvement (ROI 1–2), cost-saving and sustainability (ROI 3–4). Organisational development is foundational to organisational effectiveness and resilience [57, 58]. Thus, savings are a late return (on investment), that occur after or during sustainment. These four QI-ROI components indicate a logical progression in a QI journey. Connected to them are related concepts (e.g., productivity and efficiency), which act as mechanisms for higher level returns [16]. We consider sustainability an end ROI as it appears to be the ultimate outcome sought. However, QI programmes may continue to provide other benefits beyond this point through legacies.

In our literature review [16], over 70% of the articles highlighted sustainability as an important outcome. This was linked to adoption, spread, and dissemination of improved practices as well as QI legacies. In this study, sustainability also included the organisation itself. Sustainability is seen as holding current gains whilst continuing to evolve [59–61]. Evolution in QI is through trial-and-error. This makes stability difficult to achieve. Sustainability can be difficult to achieve for a variety of reasons [62–64]. Sustainability requires time and other resources to help embed and spread effective practices [60, 65]. Thus, sustainability includes widespread adoption of improved practices, not just the sum of multiple initiatives [59]. Sustained improvements can enhance the speed of future programmes.

As stated earlier, the speed of problem and solution identification emerged as important in this study. This is supported by how QI effectiveness is viewed in large programmes. In large programmes, effectiveness includes systemic improvements, efficient problem identification and solving [66, 67]. The speed of implementation is thought to support organisational change [68]. Paradoxically, developing the abilities for efficient problem identification and solving takes time. It follows continual learning, sustained success, and culture change [66, 67, 69]. In this process, QI programmes build a ‘QI legacy’ (retained relationships, capacities, and capabilities) and ‘QI intelligence’ (accumulated QI knowledge) that promote a ‘QI logic’ (a way of thinking about quality issues). All this can support an improvement culture where a QI philosophy is directly or indirectly infused in how new quality problems are managed. Over time, teams can more efficiently identify problems as well as identify and implement potential solutions e.g., the response to COVID-19 described by participants in this study.

In the following sections, we will discuss further some of the challenges highlighted in this study, starting with the ambiguity and uncertainty associated with QI-ROI conceptualisation.

### QI-ROI ambiguity

Ambiguity refers to the simultaneous presence of equally plausible but mutually contradictory explanations of a situation or concept [70]. This is common where multiple views co-exist [54, 71]. In this study, ambiguities presented in different mandates, values, and expectations from QI. Further some of the ambiguities appeared to be related to QI philosophy and practice. Ambiguity in QI may be a result of the amalgamation of multidisciplinary and multi-industry concepts e.g., lean and efficiency [71, 72]. When QI was introduced in healthcare, rhetoric was used to promote the QI discourse in efforts to improve communication and facilitate change [71, 72]. In this context, ambiguity can be tolerated and even desired, in-order to accommodate new ideas, multiple and diverse perspectives [73]. However, tensions and conflicts can also occur as a result. As seen in this study, QI buy-in, scepticisms, and different expectations are the remnants of this process. These factors appear to further influence how QI effectiveness and QI benefits are defined and measured, and in-turn, how QI-ROI is conceptualised.

The QI trial-and-error philosophy was one prominent example of QI philosophy ambiguity in this study. The trial-and-error mentality appear to enable conflicting meanings of success, given that QI can still be valued even if ineffective in achieving intended goals. This mentality fosters a learning environment where even negative results are seen valuable [54]. However, this also implies a paradox where failure must be seen as success. Trial-and-error aligns with the well known, ‘fail-fast philosophy that supports innovation and progress in companies such as Amazon and IBM. The fails-fast mindset is associated with Agile, an iterative software-based project management approach [74].

Although it has its criticisms, e.g., concerns about the neglect of deep-seated challenges, Agile has its advantages. The Agile philosophy empowers staff by fostering a blame-free culture [74, 75]. This can be a valuable philosophy where healthcare staff need psychological safety to experiment and innovate. It can also be an effective strategy where uncertainty about problems and solutions exist [54, 75]. As such, Lange et al., [75] suggested embracing the flexible fail-fast mentality rather that push for unattainable success. However, as seen in this study, this can also cause uncertainty about ROI where success is seen as ROI.

### QI-ROI uncertainty

Uncertainty can present in different ways depending on it’s a specific source. However, all uncertainty result from a lack of adequate information needed to make decisions [53, 76, 77]. Uncertainty impacts how QI-ROI is conceptualised by limiting perceptions about what QI is or can be beneficial for. A lack of knowledge about QI benefits, causes lack data and scientific evidence about QI-ROI. This may be compounded by differing values and assumptions about the existence, the nature or extent of QI benefits. Some uncertainty can be reduced e.g., through additional information. For example, scientific decision theory uses specific methods to reduce uncertainty depending on the source [53]. Within decision theory methods, sensitivity analysis is used in ROI analysis to assess different hypothetical scenarios [78]. However, healthcare complexity often prevents abilities to reduce uncertainty [79]. There may always be unknown unknowns or unknowable unknowns [76, 80] than require value judgements.

In traditional rational modes of thinking, value judgments may be seen as unacceptable alternatives to objective data [81]. Equally, the appropriateness of classical rational decision theories are challenged. Authors have argued that it should not be assumed that ethical and subjective decision-making is irrational [41, 55, 56, 82–85]. There is now an understanding that sensemaking is an ongoing social process, driven by plausibility rather that accuracy [56, 85, 86]. Today, managing ambiguity and uncertainty is seen as part of flexible effective leadership [73]. A lack of full scientific certainty is not seen as a reason to postpone or discontinue cost-effective interventions [76, 87]. Thus, a future QI-ROI tool must acknowledge that science alone may be insufficient to guide how to make sound reasonable judgments. Uncertainty is also compounded by the challenges in measuring and attributing externalised costs and benefits.

### Externalised QI-ROI

Due to the psycho-societal causes and impacts of some of the mental health illnesses [5, 88–90], mental healthcare frequently engages with external partners. Collaboration with external partners improves patient outcomes, and enable efficient use of resources at both organisational and systems level [91, 92]. In the future, the success of NHS trusts will be judged against their contribution to integrated care systems (ICSs), as well as their internal performance [93]. However, the tension between external and internal needs is a challenge [94]. In financial and political accounting, a societal perspective is only justified in decisions about social welfare [95]. Thus, healthcare leaders are put in conflicting stewardship positions with little guidance on how to manage their role conflict. This leads to a paradox where procedures may take precedence over accountability for performance [56]. Accountability models are however being developed [96], and the Health and Social Care Act is being updated to support integrated partnerships [97]. External costs and benefits are also hard to measure and attribute.

### Immeasurability of valued outcomes

QI’s alignment with biomedical empirical reasoning means that QI largely treats quality as a measurable property [55]. The appropriateness of quantitative approaches for judging QI effects has however been questioned [98, 99]. Today, QI evaluations can also assess qualitative implementation factors such as context and staff engagement [98]. Further, the Healthcare Financial Management Association (HFMA) and NHS Improvement provide guidance on how QI data can be collected and used to support efficiency strategies [92, 100, 101]. This intelligent data in the future may support both internal and externalised value measurement. Various measurement methods are used in mental healthcare [102], including indicators and proxies [103, 104]. However, some items remain impossible to measure, and the use of proxies and indicators is disputed for the very reason of lacking certainty [54]. Further, valid financial proxies are also lacking [105]. These challenges compound the apprehension over QI benefit monetisation.

### De-emphasis of monetisation

Healthcare has been accused of arguing ‘exceptionalism’ in relation to performance indicators used in other industries [85]. Organisations can and do measure overall financial health based on composite financial performance indicators e.g., profit, loss, cash flow, capital, ROI etc. [106]. These can then be matched against patient safety and quality indicators as well as value-based payments [106]. However, increasingly, healthcare investments are being treated as discrete and expected to produce their own ROI, e.g., ROI of IT [107], research and development [108], and leadership programmes [109]. Public services’ leaders do object to monetisation. Authors argue that the ‘bottom-line’ need not mean monetisation [105, 110, 111]. However, they are not unique in their concerns [112]. ROI is known to be one aspect of QI value [12, 30].

An economic or markets logic is blamed for being narrow and for failing to acknowledge long-term financial neglect such as that in mental healthcare [110]. Healthcare needs more resources and support to deliver both quality and save costs [113]. Opportunities to improve quality and reduce costs exist, particularly in care overuse [114]. However, the relationship between quality and cost is complex [20, 115]. The iron triangle (time, cost, quality) reflects these tensions where focusing on one aspect may compromise another [86, 116]. This tension sometimes leads to the separation of quality improvement from cost or value improvement campaigns [100]. What appears certain is that profit making is not the focus in healthcare. The NHS can however generate profit to benefit the public, and does so increasingly through commercial and private work [117, 118]. Financial outcomes do matter [119], however, increasingly non-monetary benefits are also linked to other organisational benefits, such as sustainability [120].

Healthcare is expected to engage with modern agendas such as environmental, and organisational sustainability [100, 115, 121]. In 2007, Coiera & Hovenga [68] predicted that the healthcare system will fail if it did not transform itself substantially by 2020. Such predictions spurred efforts to create sustainable healthcare organisations. Organisations must develop dynamic capabilities and capacities, use resources sustainably, and make efficiency savings [91, 100, 122]. A sustainable organisation continually meets the needs of its stakeholders [123]. This requires resilience; the ability to absorb and recover from shocks [59, 124]. However, organisational sustainability is primarily viewed in terms of financial sustainability [124, 125]. As such, a de-emphasis of monetisation in QI-ROI may be problematic. It may appear as a lack or avoidance of financial accountability.

### QI-ROI institutional logic

As noted earlier, leaders in this study conceptualised QI-ROI from a health and social care logic. It is worth noting that although the findings here appear to support the institutional theory’s assertion that organisations are driven by norms and values at the expense of efficiency and economic benefit, this is not the complete picture here. Participants in this study saw themselves as stewards of multiple stakeholders and obligations. Efficiency and financial outcomes were seen as important, but not at the expense of patients and staff outcomes. The leaders here questioned the legitimacy of the traditional ROI in their context. These concerns are shared by others, in and outside healthcare [14, 78, 84, 126–128]. The overriding logic here appeared to be driven by health and social care values. Different institutional logics may co-exist, how each logic survives depends on its centrality and compatibility with the main logic [40]. The leaders here appeared to be willing to accommodate the economic logic to a certain extent. However, more clarity is needed on the issues raised here before a conclusion is drawn.

## Reflexivity

ST’s engagement with this study and participants was preceded by prior exploration of the subject of ROI in healthcare and other industries through various literatures. This guided the choice of study design and methods. As such, the discussion on reflexivity is not to add to the rationales for the study design, but only to highlight ST’s experience and concerns during the interviews [129]. Although ST had done background reading on ROI, she had no real-life experience of what was to be discussed with the participants. As such, ST deliberately chose a curiosity-led ‘conversational’ approach due to her student status and ignorance about their lived experiences on the subject. Her intention was not merely to ‘extract information’ but to learn from participants experiences and insights.

To our knowledge, none of the participants knew ST’s background before the interviews. However, her prior knowledge did at times come through. In one interview, a participant asked ST towards the end of the interview ‘how others out there describe ROI’. ST responded by giving a brief description of ROI as ‘seen as financial returns’. In another interview, a participant remarked “…you seem to know a fair amount about ROI already”. This remark was also made at the end an interview as ST thanked the participant for what she had learnt. This also made ST self-conscious as she became concerned that she may be biasing the data she was obtaining. From that point, ST made more efforts not to appear ‘knowing’ in any way.

ST found this exercise stressful as she tried to watch her words and questions more closely. ST then became concerned that her guardedness would or was affecting the flow of the conversations. ST ultimately returned to being less guarded and allowed the conversations to flow. From there, ST’s decided to be less guarded again when and if asked about her background knowledge. In the end ST felt she had to trust that the piloted and iteratively developed topic guide was fit for purpose. That is, it will help maintain the balance between’ knowing and ignorance’ enough to permit an easy conversational flow as well as honest data gathering. That restored ST’s confidence.

## Limitations

Semi-structured questions can lead to confirmation bias resulting from phrasing or rephrasing questions as means to get specific information from participants. However, our research design acknowledged that knowledge is socially constructed. Therefore, by this virtue, we cannot rule out our influence in the data obtained. Similarly, the use of framework analysis may have limited the focus on the emergent nature of qualitative data. However, the deductive-inductive approach minimises this effect. Additionally, the sample was identified and recruited by co-author and the Trust’s QI director. This purposeful sampling method can result in sampling bias. However, the main target sample was the finite number of top-level decision-makers, who were all approached. Finally, the participants were from same Trust, which limits the diversity of views. Although views from different Trusts would provide more depth on the QI-ROI concept, the views from these participants were sufficient at this initial exploratory phase.

## Recommendations for research

Given the controversial nature of the features of ROI in mental healthcare, further clarity is needed before a conclusion can be drawn on the QI-ROI concept as it stands currently. Specifically, more data is needed on the collective view on monetisation of QI outcomes, immeasurability of valued QI benefits, the inclusion of external QI benefits, and what QI effectiveness at organisational level means. These questions may be best posed to a wider group of mental healthcare leaders to assess the prevalence and strength of these views.

## Conclusion

Mental healthcare leaders primarily conceptualise ROI as any valued benefit. Some also conceptualise ROI as a cost-saving tool. This was a result of needing to manage both the improving quality and managing scarce resources mandates. Overall, leaders sought to compromise in areas of ambiguity and uncertainty so as find a more comfortable medium to service both scarcity and quality. The strong health and social care values, as well as flexible expectations were the strong factors in maintaining the QI-ROI as a broad concept. Political pressures, health, and social care values were strong drivers for QI mandates and investment, in-spite of ambiguity and uncertainty. The unwillingness to disinvest may also be an indicator of a consistent QI-ROI concept, driven by strong health and social care philosophies.

### Supplementary Information

**Additional file 1.** 

## Data Availability

The datasets used and/or analysed during the current study are available from the corresponding author on reasonable request. Some data has been included in this published article as its supplementary documents.

## Abbreviations

QI: Quality Improvement
ROI: Return on Investment
QI-ROI: Return on Investment from healthcare quality improvement
