# Supplementary material for: How is return on investment from quality improvement programmes conceptualised by mental healthcare leaders and why: a qualitative study
Source: BMC Health Serv Res. 2023 Sep 19;23:1009. doi: 10.1186/s12913-023-09911-9 (PMC10510269; doi:10.1186/s12913-023-09911-9)
Supplement: Supplementary file 1 — Additional file 1. [file 12913_2023_9911_MOESM1_ESM.docx]

**Supplementary file 1:**

**Consolidated criteria for reporting qualitative studies (COREQ)**

**Manuscript:** How is Return on Investment from Quality Improvement perceived by Mental Healthcare Leaders and Why-A Qualitative Study.

**Source**: Tong A, Sainsbury P, Craig J. Consolidated criteria for reporting qualitative research (COREQ): a 32-item checklist for interviews and focus groups. *International Journal for Quality in Health Care*. 2007. Volume 19, Number 6: pp. 349 – 357

| **No. Item** | **Guide questions/description** | **Reported on Page #** |
| --- | --- | --- |
| **Domain 1: Research team and reﬂexivity** | | |
| *Personal Characteristics* | | |
| 1. Inter viewer/facilitator | Which author/s conducted the interview or focus group? | p. 1, line 4,  p. 28, line 915 |
| 2. Credentials | What were the researcher’s credentials? e.g., PhD, MD | p. 29, line 927 |
| 3. Occupation | What was their occupation at the time of the study? | p. 29, line 927 |
| 4. Gender | Was the researcher male or female? | p.26, lines 817-843 |
| 5. Experience and training | What experience or training did the researcher have? | p. 29, lines 927-930 |
| *Relationship with participants* | | |
| 6. Relationship established | Was a relationship established prior to study commencement? | N/A |
| 7. Participant knowledge of the interviewer | What did the participants know about the researcher? e.g., personal goals, reasons for doing the research | p. 5, lines 157-158 |
| 8. Interviewer characteristics | What characteristics were reported about the interviewer/facilitator? e.g., Bias, assumptions, reasons and interests in the research topic | p. 5, lines 161-162 |

| **Domain 2: study design** | | |
| --- | --- | --- |
| *Theoretical framework* | | |
| 9. Methodological orientation and Theory | What methodological orientation was stated to underpin the study? e.g., grounded theory, discourse analysis, ethnography, phenomenology, content analysis | p. 5, line 171 |
| *Participant selection* | | |
| 10. Sampling | How were participants selected? e.g., purposive, convenience, consecutive, snowball | p. 5, line 151 |
| 11. Method of approach | How were participants approached? e.g., face-to-face, telephone, mail, email | p.5, line 153 |
| 12. Sample size | How many participants were in the study? | p.6, line 185 |
| 13. Non-participation | How many people refused to participate or dropped out? Reasons? | p. 5, line 186 |
| *Setting* | | |
| 14. Setting of data collection | Where was the data collected? e.g., home, clinic, workplace | p.4, line 134 |
| 15. Presence of non-participants | Was anyone else present besides the participants and researchers? | N/A |
| 16. Description of sample | What are the important characteristics of the sample? e.g., demographic data, date | p.5, line 184-185 |
| *Data collection* | | |
| 17. Interview guide | Were questions, prompts, guides provided by the authors? Was it pilot tested? | p. 5, lines 163-164 |
| 18. Repeat interviews | Were repeat interviews carried out? If yes, how many? | N/A |
| 19. Audio/visual recording | Did the research use audio or visual recording to collect the data? | p. 5, line 165 |
| 20. Field notes | Were ﬁeld notes made during and/or after the interview or focus group? | N/A |
| 21. Duration | What was the duration of the inter views or focus group? | p. 5, line 166 |
| 22. Data saturation | Was data saturation discussed? | N/A |
| 23. Transcripts returned | Were transcripts returned to participants for comment and/or correction? | N/A |
| **Domain 3: analysis and ﬁndings** | | |
| *Data analysis* | | |
| 24. Number of data coders | How many data coders coded the data? | p. 5, line 158  p.29, line 918 |
| 25. Description of the coding tree | Did authors provide a description of the coding tree? | p. 6, line 176-181  Supplementary file 4 |
| 26. Derivation of themes | Were themes identiﬁed in advance or derived from the data? | p.6, line 187-189 |
| 27. Software | What software, if applicable, was used to manage the data? | p. 5, line 167 |
| 28. Participant checking | Did participants provide feedback on the ﬁndings? | N/A |
| *Reporting* | | |
| 29. Quotations presented | Were participant quotations presented to illustrate the themes/ﬁndings? Was each quotation identiﬁed? e.g., participant number. | Results; lines 205-565  supplementary file 4 |
| 30. Data and ﬁndings consistent | Was there consistency between the data presented and the ﬁndings? | Results; lines 205-565  supplementary file 4 |
| 31. Clarity of major themes | Were major themes clearly presented in the ﬁndings? | p.6, line 187-189  p.18, Figure 1 |
| 32. Clarity of minor themes | Is there a description of diverse cases or discussion of minor themes? | p. 7, lines 209-212 |

Supplementary file 2: Topic guide

How is Return on Investment from Quality Improvement perceived by Mental Healthcare Leaders and Why A Qualitative Study

**Qualitative Study Research Questions**

1. How is return on investment in Quality Improvement conceptualised?
2. What influences those conceptualisations in healthcare organisations?
3. How may these meanings influence quality improvement investment?

**Interview Questions**

These questions aim to support addressing of the above questions. They are based on the Systematic review findings and aim to ascertain in more depth how and why ROI is conceptualised a certain way by the leader and or their organisation. Further, the questions are designed to understand how that affects leader’s decision-making toward investment/disinvestment.

1a: For what objectives/goals is QI used for in your organisation?

**Question goal**:

- To ascertain leaders’ assumptions and expectations in relation to QI outcomes.

**Research question to address:**

- How is return on investment in Quality Improvement conceptualised?

1b: Why are these objectives important to the organisation?

**Question goal**:

- To ascertain leaders’ assumptions and expectations in relation to QI outcomes.

**Research question to address:**

- What influences those conceptualisations in healthcare organisations?

1c: How important are these objectives to QI investment decisions?

**Question goal:** To ascertain the significance and validity of these expectations and assumptions on the decision to invest in QI in relation to organisational needs.

**Research question to address:** How do these meanings influence the decisions that are the taken towards quality improvement investment?

2a: Do you think QI lives up to that/those objectives?

**Question goal:** To ascertain perception of QI impact

**Research question to address:**

- How is return on investment in Quality Improvement conceptualised?

2b: How do you know QI has worked to meet those objectives?

**Question goal:**

- To ascertain how they assess fulfilment of those expectations and gain understanding of consistency between assumptions, expectations, and evaluation.

**Research question to address:**

- What influences those conceptualisations in healthcare organisations? How do these meanings influence the decisions that are the taken towards quality improvement investment?

3a: What other if any benefits do you think your organisations gets from having used QI methods?

**Question goal**: To ascertain if any (other) wider internal or external organisational benefits or consideration are factored in QI ROI?

**Research question to address:** How is return on investment in Quality Improvement conceptualised?

3b: How important are these other benefits of QI to your organisation?

**Question goal:** To ascertain if these extra benefits are of any priority or significance?

**Research question to address:** How do these meanings influence the decisions that are the taken towards quality improvement investment?

4a: Are there any non-beneficial or less beneficial outcomes of QI?

**Question goal**: To ascertain what is viewed as a negative or non-beneficial QI outcome and why.

**Research question to address:** How is return on investment in Quality Improvement conceptualised?

**4b:** What do you think are the consequences of poor outcomes of QI? immediate vs longer term

**Question goal:** To ascertain if poor or negative outcomes affect future QI investment decision, if so how.

**Research question to address:** What influences those conceptualisations in healthcare organisations? How do these meanings influence the decisions that are the taken towards quality improvement investment?

5: How much of a priority do you think investing in QI is under normal and challenging times?

**Question goal:** To ascertain the extent to which the perceived value of QI is consistent and stable.

**Research question (s) to address:** How is return on investment in Quality Improvement conceptualised? What influences those conceptualisations in healthcare organisations? How do these meanings influence the decisions that are the taken towards quality improvement investment?

6a: What do you consider as investments that your organisation makes towards QI?

**Question goal:** To ascertain any consideration of wider or non-monetary investments?

**Research question to address:** What influences those conceptualisations in healthcare organisations?

6b: What do you think influences attainment of QI benefits?

**Question goal:** To ascertain perception of wider influences of organisational factors on QI?

**Research question to address:** How do these meanings influence the decisions that are the taken towards quality improvement investment?

7: What does the phrase Return-on-Investment mean to you, and how does this apply to QI?

**Question goal:** To gain insight into how the specific phrase ROI is perceived and if that has any affiliation with the other QI outcomes/benefits/consequence/value already discussed.

**Research question to address:** How is return on investment in Quality Improvement conceptualised?

8: Do you think this view is shared within your organisation?

**Question goal:** To ascertain their assumptions on the potential differences in meaning-making at an organisational level.

**Research question to address:** How is return on investment in Quality Improvement conceptualised? What influences those conceptualisations in healthcare organisations?

9: What advice would you give to NHS organisations who invested a lot in QI, and those who have not invested in QI?

**Question goal:** To summarise perceived potential benefits/outcomes of QI

**Research question to address:** What influences those conceptualisations in healthcare organisations?

**Introductory questions (on record/transcription)**

Question (i): General awareness of QI?

Can you talk to me about Quality Improvement – what does this term mean to you?

Question (ii): Role and QI decision making influence

I would like to find out a little bit about your role in this organisation.

- What it entails?
- How long you have been in this role?
- What involvement you have with QI decision-making?

Main questions and Possible probes (on record/transcription)

1a: For what objectives is QI used for in your organisation?

Possible probes:

- What problems do you expect QI to solve or improve in your organisation?

1b: How important are these objectives to the organisation?

Possible probes:

- Why are these aspects important?
- Are these objectives aligned with certain aspects of your organisation?
- Without such a focus, do you think some important aspects may be missed?

1c. Which objectives are more priority in QI investment decisions?

Possible probes:

- QI as a priority under normal circumstances?
- How would an NHS organisation decide if they should invest in QI?
- How should they decide if the investment is enough or too much?
- In your capacity as X, what do you think should matter most to the executive board when deciding how investment in QI should be renewed / cut / increased?

2. How much of a priority do you think investing in QI is or should be?

- during crises or times of limited capacity
- Is there a situation you can think of when that may not be a priority?
- e.g., funding/staff shortages, would QI still exist in current form, or at all
- Why is that so?
- Which objectives can be prioritised less?
- Has QI made a difference in the organisation’s pandemic response?

3a: Do you think QI lives up to those objectives its applied for?

- Do you believe that QI has any value or benefits for your organisation?

3b: How do you know QI has worked to meet those expectations?

Possible probes:

- Would things be different without QI?

4a. What other if any benefits do you think your organisations gets from having used QI?

Possible probes:

- Does QI affect or impact any other crucial aspects in your organisation? how?

4b. How important are these other aspects of QI benefits to your organisation?

- Why are these aspects important?
- Are these other QI aspects aligned with certain aspects of your organisation?
- Which are more important for investment decision-making?

5a. Are there any non or less beneficial outcomes of QI?

- What are the negative outcomes of QI?
- What would help the executive board know if QI is working or failing?

5b. What do you think are the consequences of poor outcomes of QI?

- What are the immediate consequences?
- What are the longer-term consequences?
- In what way are they not beneficial?
- What does these outcomes impact?
- How do these QI outcomes create problems or challenges?
- In what way does previous (if any) QI lessons contribute to?
- How do these influence investment decision-making?

6. What do you think influences attainment of QI benefits?

Possible probes:

- What do you think is necessary for your organisation to make the most of QI?
- How do you think these exert their influence?

7. What do you consider as investments that your organisation makes towards QI?

Possible probes

- Apart from financial investments, what other resources are applied to QI activities?
- Do you consider those the only or the most relevant investments?
- How important are these resources to QI payback abilities?
- How important are these resources to QI investment?

8. Are you aware of the phrase Return-on-Investment

a. The ROI process

- Have you had any experience in this process?
- What does the process involve?
- Who does the process involve?

b. ROI meaning

- What do you think this means?
- Do you think this phrase/concept represent what we have just discussed?
- In what way does it or does it not represent what we have just discussed?
- How does ROI apply to QI?
- What do you consider a QI investment payback?
- Is the idea of QI’s pay back vs objectives vs outcomes to your decision-making?
- Do you think these are different things?

9. Do you think this view is shared within your organisation?

Possible probes

- Do you think different individuals may see ROI as something else?
- Why do you think may explain these differences?

10: What advice would you give to NHS organisations who invested a lot in QI,

and those who have not invested in QI?

Possible probes:

- What do you think are the main lessons or points to consider when deciding to invest, not invest or remove investment from QI activities.

**Supplementary file 3:**

1. **How is return on investment in Quality Improvement conceptualised?**

Deductive Limb Inductive Limb

Themes parent codes child codes emerging codes

Clinical outcomes

Patient experience

Patient safety

Patient and carer engagement

Patient financial outcomes

Population outcomes

Patient and community trust

Patient outcomes

Financial outcomes

Organisational performance

Cost saving

Cost containment

Cost-reduction

~~Profit~~

Cash releasing

Investment reallocation

Awareness Skills Training Education

Experience Engagement

Safety Motivation Well-being Trust

Culture and climate

Staff outcomes

Organisational development

Team building

Team-working

Team cohesion

Team outcomes

Shared leadership

Team leadership

Leadership development

Efficiency

Productivity

Resource management

Processes and structures

Research

IT & Data

Innovation

Sustainability

Dissemination

Spread

Scaling-up

Embedding

Learning

Resilience

Legacy

Organisational development

Internal collaboration

Mission and vision

Learning

Transformation

Sustainability

Strategy Alignment

Governance

Legal

Ethical/Legal

Clinical governance

Organisational governance

External collaboration

Environment

Organisational obligations

Awards

Accreditation

Rewards

Reputation

Influence

Competitiveness

Incentives

External outcomes

Productivity

Socio-economic

Resources increase

Community/society

Safety

Sustainability

Innovation

Positive

Unintended outcomes

Top-down distortions

External imposition

High resource demands

Loss of revenue

Duplication

Loss of buy-in

Negative

Financial outcomes

ROI definitions

Non-financial

QI as a diagnostic tool

Effectiveness

Efficiency, productivity

Positive staff outcomes

Positive patient outcomes

Positive community/societal outcomes

Organisational development

Process/systems/structure improvement

Inductive Limb: New theme (s)

Themes parent codes child codes

Trial and error

Learning

Situation assessment

Situation analysis

QI as a diagnostic tool

QI methodology intent

Perceptions

ROI intent

Other’s intents

Failure to scale-up

Failure to spread

Failure to disseminate

Failure to embed

Failure to sustain

Failure to achieve intended outcomes

QI team failure

QI Failure

Achieving intended goals

Scaling up

Spread

Embedding

QI effectiveness

Supplementary file 4: codes, themes, and exemplar quotes

| Themes | Main codes | Child codes | Description | Exemplar Quotes |
| --- | --- | --- | --- | --- |
| ROI CONCEPTUALISATION |  |  | How ROI was defined or perceived by participants. | “I don't think we should ever be looking at it purely in terms of money or cost savings. I think that is a short-sighted view of what quality improvement should do. I think it should be return, I think we should change it to return on value”. **Participant 1**  “Many things in the NHS, in my experience would not be measured just on financial outcomes because you have to look at outcomes of some who people use our services, communities and families. I think we I think we have to have a wider definition of return on investment than a purely financial one”. **Participant 5**  “I'm not looking to make profits. I don't see uhm , you know, a depressed child at the end of treatment as a monetised outcome”. **Participant 11** |
|  | Any benefit |  | Different types of internal and external benefits that are a ROI. Includes patient, staff, financial, systemwide benefits, value, quality, evidence-based care. | “… a business case should be a combination. It's very simplest form, non-financial and financial benefits or a weighing up of the non-financial benefits against the financial cost. That's as a minimum. That's what there needs to be”. **Participant 1**  “the reality is that often return on investment … well my understanding is sometimes if you put money in then you will save money or save or have better processes as a result. So, it's not always about money, it's about quality of service as well”. **Participant 10**  “return investment for me means what is the impact, you know? So, you've made an investment in resources might be financial or might not be financial people, systems or whatever it is and what is the benefits? What are the benefits that you've reaped from that they can be financial or non-financial”. **Participant 4**  “ROI initiative is you look at what care are we providing at the moment… against best practice standards that achieve the best outcomes for patient ... You give them the right holistic treatment so they don't very often need to come back into your system, and they can get meaningful employment”. **Participant 2**  “I think, we in the NHS. I would put a greater store on intangible assets and what creating a spirit of enthusiasm, interest, communication, engagement, a sense of empowerment or belief we can do things. I think they're phenomenally valuable things, and they make a difference to patient care” **Participant 16** |
|  | Cost |  | Definitions of ROI that lean more heavily on cost of care | “I think some of the costs, on return on investment that could be attributed to QI, is an improvement in your experience of work. So, if a team got high turnover, causes stress and QI can fix some of those problems, then you reduce the cost does not only add paid staff off sick, but agency” **Participant 15**  “If you start to reduce the amount of turnover you getting people staying longer because they feel it's a good organisation to work. There is always a cost when someone leaves then you gotta replace them. This cost to that and there's churn and turnover, so there are some of the examples for me on return on investment”. **Participant 6**  “I think return on investment; you can measure in different ways. I mean measure it in hard terms about, we've managed to save this much money because we've reduced the number of complaints or reduce the number of incidents”. **Participant 7**  “If you're spending too much on that, you probably are not going the outcomes. That's your ROI”. **Participant 8** |
| INFLUENCING FACTORS |  |  | Factors that influence the consistency of QI-ROI conceptualisation | “for the board it will mean success and it would mean success for the public we serve, which is why we've been put in post and the positions were in so actually in reality it is just about succeeding in the roles that the public have gifted to us to by virtue of us being appointed onto”. **Participant 12**  “The board has responsibilities in law as a board of what it means to do, you know, in order to satisfy our regulators that we're doing a good enough job. Our board. It's far more ambitious”. **Participant 9** |
|  | Healthcare mandates |  | Perceived obligations and aspirations | “I have a role and accountability within the board and with the board for quality delivery. There's other delivery of the services that are safe, good outcomes, good patient experience… responsibility for oversight of the financials, but also creating an environment where we can invest appropriately in innovation, development and quality improvement” **Participant 7** |
|  |  | Main goals | The main goals which QI is geared towards. These include patients, staff, financial, system, societal outcomes improvement | “…as a board member I…I have a role and accountability within the board and with the board for quality delivery. There's other delivery of the services that are safe, good outcomes, good patient experience”. **Participant 7**  “to improve the lives and the experiences and the outcomes and realize the potential of people who use our services. But to do equally the same for the staff that are dedicated and come and work in our service”. **Participant 2**  “A commitment for our organisation to be anti-racist. A greater focus on community to support people, o live good lives, avoiding the need for admissions”. **Participant 3**  “we really interested in what are stakeholders are telling us and they might think we should be doing things very differently, but we see that fits with our strategic priorities in that we are offering equity of services to all parts of communities”. **Participant 5**  “We are making a lasting, enduring impact on people social determinants of health and their quality of life. And we want to make sure that we enhance they primary and community care offer and not go to inpatient care”. **Participant 12** |
|  |  | Objectives | The objectives that leaders wish to meet in-order to improve the stated the main organisational goals. Including sustainability, managing scarce resources. | “when I sort of talked about financial benefits. I talk about how do we then reinvest that to make us more sustainable service for the future, knowing that we've got increased demands often in decreasing capacity or capacity, that's stable…” **Participant 12**  “We've got an objective around sustainability which is about value for money. We think that this will help with our value for money because it will help too as far related to it, one of the biggest drivers for our, uh, I would, uh, overspend and deficit within the organisation is staff and the amount of bank and agency staff we use”. **Participant 6**  “there has to be a recognition that resource is limited, so it's how you maximize the benefits of your patience within a fixed resource and how you tie that back to patients. If you don't do that, what you are doing is adversely impacting patients in the future cannot access your services 'cause you ran out of money effectively or time”. **Participant 1**  “it's to get the best bang for your buck in a way you know we don't have endless money and you can't do everything, so you have to try and be as economical and productive and the highest quality to deliver the best quality of care”. **Participant 11**  “delivering a better care at lower cost and achieving, a sustainable organisation because we're working in an era of rising acuity come with, uhm, severe constraints on the money … so for me how you use the money we receive becomes critical… because it's public money. We should make sure that it's used … to maximize the benefit of to the people that we serve”. **Participant 3** |
|  | Values |  | Reasons for wanting to achieve main goals and objectives. This included intrinsic and extrinsic values. | “I know, I sounds like an old socialist and I work in a nationalized healthcare system. I kinda don't care what it costs. I care a little bit about using the best, making the best use of the time we have in our hands” **Participant 11**  “it's the right thing to do. And the UM. I think. If I've had that. And other people get that and other people from more stigmatized communities can get that. I think we will have a fantastic health service”. **Participant 15**  “most people come into the healthcare business because they want to see patients get better. They don't come in because they think we're going to shave 1000 pounds off 1 bit of a budget if we do a little bit here …” **Participant 2**  “I don't suppose many people think of it as a job as much as they think of it as being a passion and a commitment”. **Participant 9**  “I think as a mature organisation I think that and I know that having service users and carers involved in from leading their individual care …it really shows the world and us internally that we value services and cares”. **Participant 10**  “I'm conscious that often we in in the political context, we talk about the return of on investment by talking about, you know, the amount you save by the investment that you make in a new service”. **Participant 3**  “the trouble with the NHS at the moment is very focused on ‘cash releasing’ sort of things so that it drives cost out and I don't believe that that is well, it shouldn't be exclusive measure or absolutely and it may be part of it, but it is also about value, so measuring the value added and that's measuring outcomes, money on experience and money”. **Participant 7**  “I'm not looking to make profits. I don't see I, you know, uhm a depressed child at the end of treatment as a monetized outcome”. **Participant 11**  “the effort that trust should take to make sure that that the quality of services, are, is. saying and the way it's applied in the trust is make sure that frontline workers, carers. Uh, service users themselves are involved in identifying ways in which complex issues, often things that are not easily solved can be dealt with and services improved”. **Participant 5**  “we want the organisation to be a great place to work. Uh, and we're saying that to be able to be that we've got to have high levels of staff engagement”. **Participant 6** |
|  | Expectations |  | What leaders expect from QI based on leaders’ understanding of QI function relative to goals and objectives | “To my knowledge we're not where we need to be and there is room for a great deal of improvement, and certainly if you talk to people who use our services. Uhm, and talked to local communities, which we do. We know that there's a huge gap in terms of what people and what we are able to deliver the moment…” **Participant 5**  “…there's sometimes a mismatch between, the kind of high standing that the Trust has part of its research at partly to do with other links between the Trust and are all- psychiatrist, so there's other and the reality that. When you look at services within the Trust, uh, you know the outcomes are not, uh, not great. They're not certainly, if they're not kind of national leading”. **Participant 13**  “I'm not saying all of it can be stopped with QI because not everything is quality and not everything is a quality improvement project. Some of it's just about improving quality through different ways to do it, and some of it's just a one off” **Participant 15**  “…you need to, be incredibly thoughtful about where it is that you invest and focus on using that approach versus what other things you could use within the organisation, either to improve quality, improves safety, improve staff retention and wellbeing and so I would say it's worth it, but it's not a panacea to all your issues” **Participant 16**  “I think clarity of issues and diagnosis. I think QI methodology is fantastic at doing that and identification of what some of the blocks and or barriers to change and delivery will be and then crucially, what the success factors will look like. I think you can do that really quickly using QI methodology”. **Participant 12**  “that capacity of doing things differently, might not fit with a very simple kind of quality improvement of just about efficiency. It might be actually developing a new way of doing things entirely.” **Participant 13**  “just to be really clear, that doesn't mean everything QI does work. In fact, a lot of things QI does, won't work, you know. A successful business case isn't one that you get the money for the thing you wanted and do it.” **Participant 1**  “understand you're gonna have failures in it and you are gonna have failures on it and you will go backwards on some things. But don't let that put you off. You know we learn from that and then say we're going to do something in a slightly different way.” **Participant 6**  “I think the problem is, a zeal, that it solves and delivers on every problem that they're sort of that slightly religioso aspect of it, that if you only did everything with QI.” **Participant 11**  “I think that you know they did sort of a blind filter process, but I think it's really hard to do because I think everybody thinks everything's important. And of course, everybody has their own individual thing that that drives them and motivates them…”**Participant 14**  “I don't think you it can deliver organisational transformational change a, an infrastructure of an entire trust. I don't think it can do that.” **Participant 9** |
|  | Ambiguity |  | Several ambiguities within and between their understanding of QI function, success, measurability, and monetisability. | “I would only ever on the list if we could measure it and that would be with and you left us some sort of psychological safety type so beginning and end for service, for staff and for service users. But we still add a narrative”. **Participant 15**  “not immediately obvious in money. If you if you want, you can probably drill it down some money, but just from a a broad-brush stroke people wouldn't immediately”. **Participant 14**  “I think they're sort of Holy Grail of outcomes. If you like that healthcare has to cover. Some of those you can translate into financial metrics”. **Participant 4**  “I think probably now we don't think of it so much like that because it's actually embedded in the organisation. It's not an ad on, it's business as usual. It's what we do. So, It's part of us. So, in the same way you would say well how, how do you measure the investment of investment we've made in the HR department”. **Participant 9**  “this quality improvement is something that you invest in, but it doesn't give you anything for a while. You know you've got. You might have to wait two years before you get anything you know you gotta be patient…” **Participant 1**  “I think there is an expectation that it should be driving out those that that ROI and that benefits sort of almost immediately after each project is run”. **Participant 4**  “I think there's a slight split brain, [the] organization is gone or well, I've just invested 10 million in QI. So, like you know, come on, where's my results? Where's my results? And you know, obviously, that's not what it's about at all. It's about an ethos, a way of looking at things, the way of thinking about things, creating huge infrastructure you need in order to be able to even just look at your data”. **Participant 16**  “do I use return on investment to mean all of the benefits that accrue from QI. Yes. Should I not be so lax about that? Probably also the case because I can see legitimately why we might want to say ROI is the bit-that's the financial element of it, and sometimes it gets lost alongside the softer benefits, so I can see that there's a reason why you'd want to focus on the financial benefits. But in my loose language, do I equate benefits to ROI, yes? I probably do”. **Participant 4**  “So I think that people often say QI and don't know what they mean, so that people use it and potentially not be trained or understand how to use CQI or continuous improvement methodologies and therefore not implemented properly and then not like it because they don't think it doesn't work”. **Participant 12**  “I think they want results, and they want things changed quickly. ..they want is an instant change, and it doesn't happen…I mean, I think there's a bit of lack of understanding”. **Participant 15** |
|  | Uncertainty |  | Expressions of uncertainty over QI outcomes or ROI. These include issues with causality, and poor communication regarding outcomes. | “…there is absolutely no guarantee that they will deliver”. **Participant 3**  “one of my frustrations would be that I think it's hard to see the return on investment from QI at the moment. I, I think that would be something that would be a common comment from people if you ask them, but from where I sit, I think it's hard for me to see explicitly what the return on investment is” **Participant 1**  “I'm not in a position to say that and I would add though, the point I made earlier, which was a criticism from, someone working in our trust that too often, quality improvement projects fizzle out”. **Participant 8**  “I think there's more to be done around the impact, so we got a lot of involvement. I think maybe QI could help with what the impact is and sort of looking at the methodology around impact”. **Participant 10**  “I'm uncertain if I could really answer that. If at the time when we were initially sort of rolling out and embedding, if we'd ask patients what it meant to them, they probably, I don't think they would have particularly said anything, and one of the key agendas was to include service users from the get-go in new project design and your ideas, and we did”. **Participant 14**  “personally think that at the minute we've got some exceptional QI programs where people have improved elements. I think there's still quite a lot of I would say uncertainty about at, say, an organisational level, a big improvement program, what's going to work”. **Participant 2**  “we know we're certainly seeing benefits of the things that we've done through QI. I'm not sure we're very good at advertising it”. **Participant 9**  “I think with anything, you can never 100% stand there and say this is directly attributable to X, but you might be able to conclude to the best of your working knowledge”. **Participant 14**  “…staff satisfaction, how do I measure that? I can see their measure, but I can't put a monetary value on it, but I know from all of the evidence that we that we have”. **Participant 6**  “I don't know whether we would that easily, I think we would uhm as we sit at the moment, we would struggle to measure it up as clearly and systematically as we would have liked…” **Participant 7**  “it's quite complicated to measure… the danger is that the upfront costs are kind of easier to measure and the benefits are probably harder to measure, so it's a challenge to demonstrate that kind of return on investment just because I, I think that the benefits are harder to measure, probably”. **Participant 13**  “It's always about what the measurable outcomes of the improvement you're making. And then there's other people that often think about how do I then translate that into a return on investment or a financial projection for organisation? And that's often what the board job is-I think to the hold that uncertainty and try to bring that level of clarity without putting that burden on staff that, may not be at that level of experience or understanding” **Participant 12** |
| DISINVESTMENT POTENTIAL |  |  |  | “I think I think in order to understand the financial impact of that investment that they have made to you know. So sometimes you make decisions based on the fact that you won't get any of this. You know you won't get any financial returns. Sometimes you make it based on the fact that actually you’ll save”. **Participant 4**  it's quite resource intensive to start with and so your returns come over longer periods of time. As I say, they're not cash releasing. As the pressure on the money increases. You got under pressure that you start reducing the investment or not investing more when you need to or and not in particular areas and the pressure in the service as a whole means people are just so hard pressed that it it falls to the bottom of the list of immediate priorities. **Participant 7** |
|  | Low |  | low expressions of wishes to disinvest from QI | “we have invested in a quality centre which oversees all our all our major QI initiatives. So, you know we are committed to QI, certainly for the moment”. **Participant 5**  “if we then said no, no, we're going to stop all this, 'cause we're just doing this. I think we wouldn't learn. We actually would miss some things as well”. **Participant 6**  “there's [been] some noise about whether or not it's been as effective as it could be. And you know what's gone wrong with QI, but it's never translated into the disinvestment”. **Participant 9** |
|  | Moderate |  | Concerns about QI but wishes to re-examine QI rather than disinvest when QI is ineffective | “I don’t think they would, but it's not really about QI methodology, it's more about the whether people are practicing that way of working, uhm deliver in a timescale that's needed by the organisation.” **Participant 12**  **“**I think if nothing got better. I think if you've applied it properly, to strategic or like significant issues., and you're not getting either the improvements in outcomes or the engagement and staff. You need to do something different.” **Participant 16**  “if there are whole host projects which are consuming staff time which are not geared to these strategic priorities for the board, then that is a problem, some of those projects would have to stop.” **Participant 5**  “if you don't do it right, you're gonna struggle to get money to invest in it because you know, we, you know; private sector allows shareholders to do it, we've got taxpayer responsibility.” **Participant 6**  “so I might change my view on how you go about QI, as opposed to stopping altogether, so it might be smaller scale, or it might be much more focused on one or two key objectives…”**Participant 7**  “it wouldn't say we're going to stop the program and disinvest from the program. It would merely node be a quite a difficult discussion about well, what's going on, what we need to change, and how we're going to turn that round.” **Participant 8**  “I would struggle to think in an ever-changing NHS that anyone has got it so completely nailed that they don't need that support anymore.” **Participant 9** |
|  | High |  | low expressions of wishes to disinvest from QI | I would say I don't think there are enough measurable examples that I am aware of, that would justify QI in our organization at the moment, and again, that's the difference between me believing in it fundamentally as a concept, which I do. and believing it is a mechanism used by my organization. **Participant 1**  “I think there is a need, to move away from QI with Capital QI as a brand, and support all members that work in healthcare systems to have a toolkit of approaches, including audit, you know, whatever, … standards appraisal”. **Participant 16** |
